# Lung cancer in the socioeconomic and environmental context of the Amazon region of Pará

**DOI:** 10.1590/1980-220X-REEUSP-2026-0036en

**Published:** 2026-07-31

**Authors:** Bruna Rafaela Leite Dias, Laura Maria Vidal Nogueira, Ivaneide Leal Ataíde Rodrigues, Eliã Pinheiro Botelho, Bruna Puty, Maridalva Ramos Leite, Gracileide Maia Corrêa, Altem Nascimento Pontes

**Affiliations:** 1 Universidade do Estado do Pará, Centro de Ciências Biológicas e da Saúde, Programa de Pós-Graduação em Enfermagem, Belém, PA, Brazil.; 2 Universidade Federal do Pará, Instituto de Ciências da Saúde, Programa de Pós-Graduação em Enfermagem, Belém, PA, Brazil.; 3 Faculdade Integrada da Amazônia, Belém, PA, Brazil.; 4 Universidade do Estado do Pará, Centro de Ciências Biológicas e da Saúde, Departamento de Enfermagem Comunitária, Belém, PA, Brazil.; 5 Universidade do Estado do Pará, Centro de Ciências Naturais e Tecnologia, Programa de Pós-Graduação em Ciências Ambientais, Belém, PA, Brazil.

**Keywords:** Noncommunicable Diseases, Lung Neoplasms, Spatial Analysis

## Abstract

**Objective:**

To analyze the incidence of lung cancer associated with social, economic, and environmental indicators.

**Method:**

This is an ecological study with 700 new cases of lung cancer, extracted from the IntegradorRHC-INCA database. Average annual incidence rates were calculated, adjusted using the direct method, and subjected to geographically weighted regression analysis. The independent variables were socioeconomic and environmental indicators, including per capita income and primary healthcare coverage.

**Results:**

A positive correlation was observed between incidence and per capita income, as well as with primary healthcare coverage.

**Conclusion:**

The results point to the need for targeted interventions and the strategic allocation of resources in priority areas, as well as the expansion and strengthening of Primary Health Care for early detection, prevention, and guidance on the risks of developing lung cancer.

## INTRODUCTION

Cancer is a major public health problem, which has a strong relationship with socioeconomic factors, especially in the 21st century, being responsible for approximately one in six premature deaths (16.8%) and one in four deaths (22.8%) due to chronic non-communicable diseases (NCDs) worldwide^(1)^. With nearly 2.5 million new cases and more than 1.8 million deaths worldwide, lung cancer was the leading cause of cancer morbidity and mortality in 2022, ranking first among men and second among women^(2)^.

In this context, in Brazil, lung cancer was the deadliest cancer in 2020 and is estimated to be one of the most frequent cancers between 2023 and 2025, along with breast and prostate cancers^(3)^. Furthermore, according to projections of age-standardized global incidence rates, it is believed that these rates will continue to increase dramatically among women in most countries until 2035, including Brazil^(4)^.

From this perspective, the expected number of new cases of trachea, bronchi, and lung cancer in Brazil for each year of the three-year period from 2023 to 2025 is 32,560 cases, corresponding to an estimated risk of 15.06 cases per 100,000 inhabitants in the year 2023. During the same period, the state of Pará, territory of the Legal Amazon, presented an incidence rate of 7.25 cases per 100,000 inhabitants^(5)^.

The social determinants of health, considered factors that directly influence the health levels of the population, including housing, water supply and wastewater services, environment, work, income, among others^(6)^, are noticeable. These determinants, in turn, are associated with the risk of illnesses and with lung cancer treatment outcomes^(7)^, and influence the distribution of the condition in space^(2)^.

Given the widespread registration of cancer cases across the country, the Brazilian Ministry of Health has been improving its actions of prevention, screening, diagnosis, and treatment supported by the National Policy for Cancer Prevention and Control (*PNPCC*) and implemented within the Health Care Network (*RAS*), structured with seven components: Primary Care; Home Care; Specialized Care; Support Systems; Regulation; Logistics Systems; and Governance^(8)^. In Primary Care, case tracking occurs due to the integration of health teams into the territories.

Therefore, to better understand the relationship between cancer and the dynamics of geographic space, techniques for spatial analysis were identified. In Portugal, for example, the geographically weighted regression (GWR) was used to investigate the relationship between the relative risk of lung cancer mortality and air pollution^(9)^, proving to be a robust technique.

Nonetheless, evidence from a literature review assessing the incidence of lung cancer using spatial analysis methods indicated that GWR was not used to analyze the influence of socioeconomic and environmental indicators on new cases of the disease, thus limiting the results obtained. It is worth mentioning that the use of GWR models allows for a more detailed visualization of the problem, according to each region studied, since it indicates spatial dependence, when applicable^(10)^.

Given this gap, this study aims to analyze the incidence of lung cancer associated with social, economic, and environmental indicators. The central question is to understand how these determinants influence the geographical distribution of the disease, based on the hypothesis that there is significant spatial dependence between the indicators and the incidence of lung cancer, which may reveal territorial patterns relevant to health planning.

## METHOD

### Design of Study

This is an ecological study with a multiple-group design.

### Context

The state of Pará is located in the North region of Brazil, with a population of 8,120,131 inhabitants and a territorial area of 1,245,870.704 km^2^. In the national ranking, it occupies the 24th position in the Human Development Index (HDI), with a value of 0.646, and the 20th position in the Social Vulnerability Index (SVI), with a value of 0.299^(11)^. The state is comprised of 144 municipalities, but has only four healthcare facilities authorized to provide specialized and comprehensive care to cancer patients, including diagnosis and treatment^(12)^.

### Participants

The study included 700 new cases of lung cancer reported between January 1, 2017, and December 31, 2021. All cases originate from one of the municipalities in the state of Pará and were obtained through the IntegradorRHC-INCA system, which gathers hospital data from cancer registries.

### Variables

The variables analyzed were grouped into socioeconomic, environmental, and health structure categories. Among the socioeconomic variables, the following stand out: Municipal Human Development Index (MHDI), Gross Domestic Product (GDP), Social Vulnerability Index (SVI), Gini Index, per capita income, illiteracy rate, basic healthcare coverage, and the proportion of people living in poverty, extreme poverty, and those vulnerable to poverty. The environmental variables included the Air Quality Index (AQI), percentage of natural vegetation cover, hotspots, and increase in deforestation. Variables related to the labor market were also considered, such as the percentage of employed individuals in the agricultural, mining, manufacturing, industrial public utility services, construction, commerce, and service sectors.

### Data Source/Measurement

Population and cartographic data were obtained from the Brazilian Institute of Geography and Statistics (IBGE), including the database for the 144 municipalities in the State. Average annual lung cancer incidence rates were calculated by municipality and adjusted for age group using the direct method^(13)^ based on the 2022 demographic census. The Microsoft® Excel® for Microsoft 365 MSO software was used for the calculations. The socioeconomic variables were extracted from the Atlas of Human Development in Brazil (AtlasBR) and the *e-Gestor* AB platform of the Ministry of Health. The environmental variables were obtained from the platforms TerraBrasilis, *Programa Queimadas* (INPE), and the AccuWeather application. The density of healthcare facilities authorized to provide cancer care was calculated based on data from the National Cancer Institute (*INCA*).

### Bias

As this is an ecological study based on aggregated secondary data, there are limitations regarding the accuracy of the individual records. The possibility of underreporting, inconsistencies in the data, or the absence of specific information can introduce bias into the analysis. Furthermore, the use of aggregated data by municipality prevents direct inferences about individuals.

### Study Size

The study included 700 new cases of lung cancer registered in the state of Pará over five years.

### Quantitative Variables

Incidence rates were expressed by municipality and adjusted for age group. The socioeconomic and environmental indicators were considered in their numerical forms.

### Statistical Methods

An initial collinearity analysis was performed between the dependent variable, the age-adjusted average annual incidence rate of lung cancer, and the independent variables using Pearson’s correlation, using Minitab 22.1 software. Subsequently, multicollinearity among the independent variables was assessed using the Variance Inflation Factor (VIF), also in Minitab 22.1. VIF values greater than 10 were considered indicative of significant multicollinearity, and such variables were excluded from the subsequent model.

Next, the “stepwise” technique was applied in Geoda 1.14.0 software to select the most relevant variables, resulting in an ordinary least squares (OLS) linear regression model with the lowest Akaike Information Criterion (AIC). The model was evaluated for the intercept value, local β coefficients, p-value, coefficient of determination (R^2^), and adjusted coefficient of determination (adjusted R^2^). The selected variables were then used to construct the GWR model, and the results were represented by means of thematic maps created in ArcGIS® 10.6 software.

### Ethical Aspects

As this study used publicly available secondary data, review by a Research Ethics Committee was not required.

## RESULTS

The study analyzed 700 new cases of lung cancer. Table 1 presents the statistical analysis of socioeconomic and environmental indicators based on Pearson’s correlation, which ranged from -0.211 to 0.265.

**Table 1:** Correlation between socioeconomic and environmental indicators and age-adjusted lung cancer incidence – Belém, PA, Brazil (2024).

Variable	Pearson correlation	p value
Municipal HDI	0.264	0.001
Per capita income	0.265	0.001
Basic healthcare coverage	0.239	0.004
Percentage of natural vegetation cover	-0.211	0.011
Percentage of those employed in the agricultural sector	-0.171	0.041
Percentage of those employed in the industrial public utility services sectors	0.192	0.021
Percentage of those employed in the construction sector	0.160	0.056
Percentage of those employed in the trade sector	0.188	0.024
Percentage of those employed in the service sector	0.199	0.017
Proportion of poor people	-0.197	0.018
Proportion of extremely poor people	-0.191	0.022
Proportion of people vulnerable to poverty	-0.174	0.037
Percentage of those employed in the manufacturing industry	0.057	0.497
Percentage of those employed in the mining sector	-0.022	0.790
Illiteracy rate	-0.149	0.074
Hotspots	0.078	0.353
Gini Index	0.018	0.828
Increased deforestation	-0.047	0.576
AQI	-0.040	0.631
SVI	0.014	0.872
GDP	0.088	0.294
Density of authorized establishments	0.144	0.085

Regarding the dependent variable, the adjusted average annual incidence rate of lung cancer, the variables MHDI, per capita income, basic healthcare coverage, percentage of natural vegetation cover, percentage of those employed in the agricultural sector, percentage of those employed in the industrial public utility services sector, percentage of those employed in the construction sector, percentage of those employed in the commerce sector, percentage of those employed in the services sector, proportion of those living in poverty, and proportion of those vulnerable to poverty showed statistical significance. Multicollinearity analysis using VIF indicated that the variables per capita income and primary care coverage had values below the cutoff point of 10, showing no significant multicollinearity between the predictors and allowing their inclusion in the following model.

The OLS model presented an adjusted R^2^ that explained only 9.93% of the variability in the average annual age-adjusted lung cancer incidence rates during the period. Per capita income and primary care coverage remained as predictor variables (Table 2).

**Table 2:** OLS model for age-adjusted lung cancer incidence – Belém, PA, Brazil (2024).

Variables	Estimate	Standard error	p value
Constant	–0.0495123	0.0746433	0.50821
Per capita income	0.000476318	0.00016111	0.00365
Basic healthcare coverage	0.00208483	0.000806072	0.01071

R^2^ = 0.111; Adjusted R^2^ = 0.099.

p-value of the model < 0.001.

It is observed that the coefficient estimate for per capita income and for primary care coverage is positive and statistically significant, suggesting that an increase in these two variables is associated with an increase in lung cancer incidence rates.

**Figure 1:**
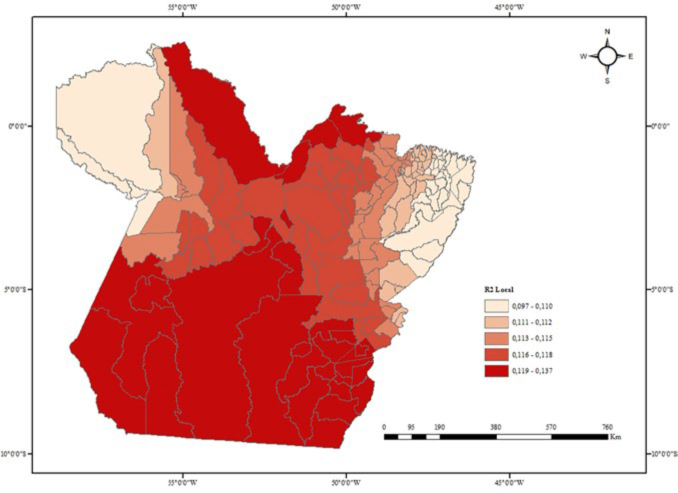
Spatial distribution of the local R^2^ of the geographically weighted regression for age-adjusted lung cancer incidence – Belém, PA, Brazil (2024).

After defining the OLS model, GWR was used, employing a fixed band for better model fit, confirmed by the lower AIC (fixed band = –7.684; adaptive band = –7.385). With this model, an R^2^ = 0.136 and adjusted R^2^ = 0.107 were obtained, with the local R^2^ varying from 0.09 to 0.13 (Figure 1).

**Figure 2:**
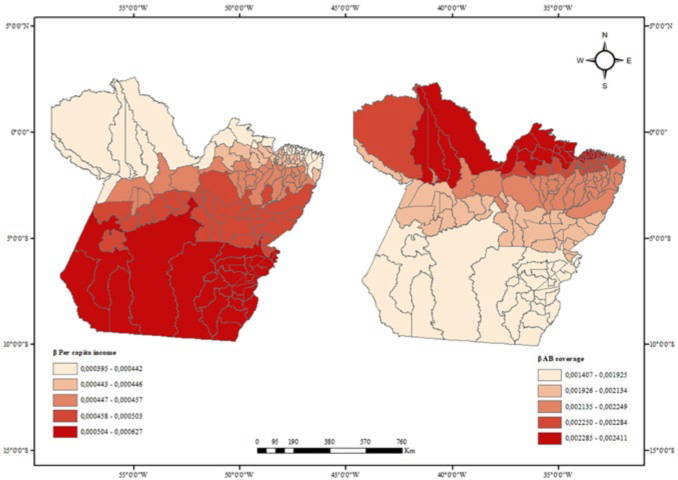
Spatial distribution of local β coefficients of independent variables in the geographically weighted regression for age-adjusted lung cancer incidence – Belém, PA, Brazil (2024).

Figure 2 shows the spatial distribution of the local β coefficients of the independent variables that made up the model. The local β coefficients for per capita income indicate considerable variations in different regions, with areas in the Southwest and Southeast of Pará showing higher positive coefficients, suggesting a stronger association between per capita income and the incidence rate in these locations.

Conversely, the local β coefficients of the variable “primary care coverage” demonstrated that in mesoregions, such as Baixo Amazonas, Marajó and Metropolitana de Belém, the relationship is more pronounced, reflecting a greater influence on the incidence of lung cancer due to the opportunity for greater access to diagnosis.

## DISCUSSION

This study showed that the incidence of lung cancer is autocorrelated in the state of Pará, with per capita income being one of the predictors of the event.

A similar result was observed in another Brazilian study, which analyzed the behavior of lung cancer in urban centers, identifying a positive association with per capita income for both advanced-stage diagnosis and lung cancer mortality. This relationship was evident in territories with higher development indices, older populations, and better access to healthcare services^(14)^.

This occurs because per capita income has a strong influence on lifestyle choices related to health promotion, as evidenced in practices such as physical activity, self-management, nutrition, and health responsibility^(15)^, resulting in a higher life expectancy. Furthermore, with higher income, there is more investment in health education, a possible reduction in the risks of smoking, and recognition of the importance of regular checkups, contributing to the early detection of lung cancer^(16)^.

It is also worth mentioning that in territories with higher per capita income, there is better access to health services, including early diagnosis and advanced treatment^(17)^. Of particular note here is Primary Health Care (PHC), whose positive variations in coverage, in this study, were also identified as predictors for the increased incidence of lung cancer in the state of Pará.

Given socioeconomic development, the need for better coordination of healthcare emerges. In view of this, primary care, as the first level of care, acts as a coordinator and organizer of *RAS*, providing preventive care and interventions to manage the burden of CNCDs, including lung cancer. As the preferred entry point to *RAS*, PHC plays a critical role in early diagnosis and referral to other levels of care^(18)^.

In this regard, in the United States, the US Preventive Services Task Force (USPSTF) recommends that patients between 50 and 80 years of age, with a 20-year smoking history, who currently smoke or have quit smoking within the last 15 years, undergo annual low-dose computed tomography (LDCT) scans for lung cancer^(19)^. However, about 50% of those eligible for LDCT, according to the USPSTF recommendations, are uninsured or covered by Medicaid, which in many states does not cover the test^(20,21)^.

Furthermore, still in the aforementioned country, there are other obstacles to the diagnosis of lung cancer^(19)^. Residents of rural areas, although more affected by smoking and the incidence of the disease compared to urban areas, tend to have inadequate insurance coverage and face geographical barriers to access^(22,23)^. Therefore, they are more likely to have limited access to primary care professionals who would recommend LDCT and refer them to specialized care when necessary^(24)^.

In this US context, it was found that low health insurance coverage was reflected in lung cancer screening, as well as in the diagnosis of asthma and other respiratory diseases. The expansion of recommended screening was significant among high-risk men, but not among women at the same risk. There are some gaps in patient awareness of the option to be screened, such as those regarding knowledge of the benefits of the test, stigma related to smoking, and lack of access to care regarding the contributing factors to low female adherence^(25)^.

In Pará, although there are significant challenges to achieving ideal coverage, between 2010 and 2021 there was progressive growth in primary health care coverage through the Family Health Strategy (FHS)^(26)^. This fact may justify the findings of this study, since with broader primary care coverage, there are more opportunities for early disease detection and access to diagnostic tests for lung cancer.

Considering the nature of this study, the ecological fallacy is acknowledged as a possible limitation of the research, and the results cannot be considered at an individual level. Furthermore, secondary data is used, which may introduce information bias due to the quality of data entry. In addition, the complexity of the GWR model can lead to difficulties in its application and interpretation.

However, the application of geographically weighted spatial regression allowed for a more detailed and localized view of the relationships between variables, since, unlike traditional regression models, it considers local geographic variation, as a pattern applied to one area does not necessarily apply to others^(27)^. For nursing, this represents an opportunity for more strategic action, focusing on priority territories and vulnerable populations, promoting equity in access to and quality of care.

Finally, the findings reinforce the importance of the connection between epidemiological surveillance, territorial management, and clinical practices. Nursing, as the majority workforce in the Brazilian Public Health System, is positioned to lead initiatives in prevention, education, and comprehensive care, contributing to the reduction of morbidity and mortality from lung cancer and to the strengthening of the healthcare network.

## CONCLUSION

The results of this study show that, in the state of Pará, especially in the metropolitan region of Belém, the incidence of lung cancer directly reflects socioeconomic development and the structure of the healthcare network. Higher per capita income and greater PHC coverage were associated with increased disease detection, indicating that early diagnosis and access to specialists are facilitated in territories with better socioeconomic conditions and a more organized healthcare network.

Therefore, it becomes essential to direct strategic interventions and resources to increase equity, strengthening PHC as the gateway for prevention, screening, and timely referral. By expanding coverage and improving the quality of services, it will be possible to reduce regional inequalities and promote a greater impact on lung cancer morbidity and mortality.

This provides a solid foundation for future research to delve deeper into the impact of PHC on the prevention and control of lung cancer, particularly studies demonstrating the economic impact of the disease and effective ways to utilize resources.

## Data Availability

All the data supporting the results of this study were published in the article itself.

## References

[1] Bray F, Laversanne M, Weiderpass E, Soerjomataram I (2021). The ever-increasing importance of cancer as a leading cause of
premature death worldwide.. Cancer.

[2] Bray F, Laversanne M, Sung H, Ferlay J, Siegel RL, Soerjomataram I (2024). Global cancer statistics 2022: GLOBOCAN estimates of incidence
and mortality worldwide for 36 cancers in 185 countries.. CA Cancer J Clin.

[3] Santos MO, Lima FCS, Martins LFL, Oliveira JFP, Almeida LM, Cancela MC (2023). Estimated cancer incidence in Brazil, 2023–2025.. Rev Bras Cancerol.

[4] Luo G, Zhang Y, Etxeberria J, Arnold M, Cai X, Hao Y (2023). Projections of lung cancer incidence by 2035 in 40 countries
worldwide: populationbased study.. JMIR Public Health Surveill.

[5] Brasil. Ministério da Saúde. (2022). Instituto Nacional de Câncer.. Estimativa 2023: incidência de câncer no Brasil [Internet].

[6] Brasil. Lei nº 8.080 de 19 de setembro de 1990. Dispõe sobre as condições para a promoção, proteção e recuperação
da saúde, a organização e o funcionamento dos serviços correspondentes e dá
outras providências [Internet].. 19 set 1990.

[7] Yu Z, Yang X, Guo Y, Bian J, Wu Y (2022). Assessing the documentation of social determinants of health for
lung cancer patients in clinical narratives.. Front Public Health.

[8] Brasil. Portaria de Consolidação nº 2 de 28 de setembro de
2017. Consolidação das normas sobre as políticas nacionais de saúde do
Sistema Único de Saúde [Internet].. 28 set 2017.

[9] Cardoso D, Painho M, Roquette R (2019). A geographically weighted regression approach to investigate air
pollution effect on lung cancer: A case study in Portugal.. Geospat Health.

[10] Arcêncio RA, Belchior AS, Arroyo LH, Bruce ATI, Santos FL, Yamamura M (2022). Distribuição e dependência espacial da mortalidade por
tuberculose em um município da região amazônica.. Cad Saude Colet.

[11] Instituto Brasileiro de Geografia e Estatística. (2023). Panorama do censo 2022 [Internet].. https://censo2022.ibge.gov.br/panorama/.

[12] Brasil. Ministério da Saúde. (2022). Instituto Nacional de Câncer.. Onde tratar pelo SUS [Internet].

[13] Rothman KJ, Greenland S, Lash TL (2009). Modern epidemiology..

[14] Lima KYN, Cancela MC, Souza DLB (2022). Spatial assessment of advanced-stage diagnosis and lung cancer
mortality in Brazil.. PLoS One.

[15] Liu Q, Huang S, Qu X, Yin A (2021). The status of health promotion lifestyle and its related factors
in Shandong Province, China.. BMC Public Health.

[16] Li C, Lei S, Ding L, Xu Y, Wu X, Wang H (2023). Global burden and trends of lung cancer incidence and
mortality.. Chin Med J (Engl).

[17] Souza GD, Junger WL, Silva GA (2019). Lung cancer mortality trends in different urban settings in
Brazil, 2000–2015.. Epidemiol Serv Saude.

[18] Hou X, Liu L, Cain J (2022). Can higher spending on primary healthcare mitigate the impact of
ageing and non-communicable diseases on health expenditure?. BMJ Glob Health.

[19] Krist AH, Davidson KW, Mangione CM, Barry MJ, Cabana M, Caughey AB (2021). US preventive services task force recommendation statement.

[20] Gomes R, Nederveld A, Glasgow RE, Studts JL, Holtrop JS (2023). Lung cancer screening in rural primary care practices in
Colorado: time for a more team-based approach?. BMC Prim Care.

[21] American Lung Association. (2021). State of lung cancer 2021 Report [Internet].. https://www.lung.org/getmedia/ba972351-ddc5-46b2-8e0d-028002d16c72/solc-2021-print-report-final.pdf.

[22] Reihani AR (2021). Barriers and facilitators to lung cancer screening in the United
States: a systematic review of the qualitative literature.. J Health Soc Sci.

[23] Le T, Miller S, Berry E, Zamarripa S, Rodriguez A, Barkley B (2022). Implementation and uptake of rural lung cancer
screening.. J Am Coll Radiol.

[24] Rivera MP, Katki HA, Tanner NT, Triplette M, Sakoda LC, Wiener RS (2020). Addressing disparities in lung cancer screening eligibility and
healthcare access.. An Official American Thoracic Society Statement.

[25] Sun J, Perraillon MC, Myerson R (2022). The impact of medicare health insurance coverage on lung cancer
screening.. Med Care.

[26] Pará. Agência Pará. (2021). Descentralização dos serviços de saúde, com investimentos do
Estado, fortalece o SUS no Pará [Internet].. https://www.saude.pa.gov.br/descentralizacao-dos-servicos-de-saude-com-investimentos-do-estado-fortalece-o-sus-no-para/.

[27] Gutierrez L, Sassi M (2012). Spatial and non spatial approaches to agricultural convergence in
Europe.. Econ Dirit Agroaliment [Internet].

