# Supplementary material for: Lung cancer in the socioeconomic and environmental context of the Amazon region of Pará
Source: Rev Esc Enferm USP. 2026 Jul 31;60:e20260036. doi: 10.1590/1980-220X-REEUSP-2026-0036en (PMC13432011; doi:10.1590/1980-220X-REEUSP-2026-0036en)
Supplement: Translation

# Translation: Câncer de pulmão no contexto socioeconômico e ambiental da Amazônia paraense

**DOI:** 10.1590/1980-220X-REEUSP-2026-0036pt

**Authors:** Bruna Rafaela Leite Dias, Laura Maria Vidal Nogueira, Ivaneide Leal Ataíde Rodrigues, Eliã Pinheiro Botelho, Bruna Puty, Maridalva Ramos Leite, Gracileide Maia Corrêa, Altem Nascimento Pontes

**Affiliations:** 1 Universidade do Estado do Pará, Centro de Ciências Biológicas e da Saúde, Programa de Pós-Graduação em Enfermagem, Belém, PA, Brasil.; 2 Universidade Federal do Pará, Instituto de Ciências da Saúde, Programa de Pós-Graduação em Enfermagem, Belém, PA, Brasil.; 3 Faculdade Integrada da Amazônia, Belém, PA, Brasil.; 4 Universidade do Estado do Pará, Centro de Ciências Biológicas e da Saúde, Departamento de Enfermagem Comunitária, Belém, PA, Brasil.; 5 Universidade do Estado do Pará, Centro de Ciências Naturais e Tecnologia, Programa de Pós-Graduação em Ciências Ambientais, Belém, PA, Brasil.

**Keywords:** Doenças não transmissíveis, Neoplasias pulmonares, Análise espacial

## Abstract

**Objetivo:**

Analisar a incidência de câncer de pulmão associada a indicadores sociais, econômicos e ambientais.

**Método:**

Trata-se de um estudo ecológico com 700 casos novos de câncer de pulmão, extraídos do IntegradorRHC-INCA. Foram calculadas as taxas médias anuais de incidência, que foram ajustadas pelo método direto e submetidas à análise de regressão geograficamente ponderada. As variáveis independentes foram os indicadores socioeconômicos e ambientais, entre elas a renda per capita e a cobertura de atenção básica.

**Resultados:**

Observou-se relação positiva entre a incidência e a renda per capita, assim como com a cobertura de atenção básica.

**Conclusão:**

Os resultados apontam para a necessidade de intervenções direcionadas e a alocação estratégica de recursos em áreas prioritárias, assim como a expansão e o fortalecimento da Atenção Primária em Saúde para a detecção precoce, prevenção e orientações sobre os riscos para adoecimento por câncer de pulmão.

## INTRODUÇÃO

O câncer é um importante problema de saúde pública, que apresenta forte relação com fatores socioeconômicos, sobretudo no século XXI, sendo responsável por aproximadamente uma em cada seis mortes prematuras (16,8%) e uma em cada quatro mortes (22,8%) por doenças crônicas não transmissíveis (DCNT) em todo o mundo^(1)^. Com quase 2,5 milhões de novos casos e mais de 1,8 milhões de mortes em todo o mundo, o câncer de pulmão foi a principal causa de morbidade e mortalidade por câncer em 2022, ocupando o primeiro lugar entre os homens e o segundo entre as mulheres^(2)^.

Nesse contexto, no Brasil, o câncer de pulmão foi o mais mortal em 2020, sendo estimado como um dos mais frequentes no período de 2023 a 2025, assim como os cânceres de mama e de próstata^(3)^. Outrossim, conforme previsões das taxas globais de incidência padronizadas por idade, acredita-se que estas continuem a aumentar drasticamente entre as mulheres, na maioria dos países até 2035, inclusive no Brasil^(4)^.

Nessa perspectiva, o número previsto de casos novos de câncer de traqueia, brônquios e pulmão para o Brasil, para cada ano do triênio de 2023 a 2025, é de 32.560 casos, correspondendo ao risco estimado de 15,06 casos por 100 mil habitantes no ano de 2023. No mesmo período, o estado do Pará, território da Amazônia Legal, apresentou taxa de incidência igual a 7,25 casos para cada 100 mil habitantes^(5)^.

Destacam-se os determinantes sociais da saúde, considerados fatores que influenciam diretamente os níveis de saúde da população, incluindo moradia, saneamento básico, meio ambiente, trabalho, renda, entre outros^(6)^. Estes determinantes, por sua vez, estão associados ao risco de adoecimento e aos resultados do tratamento do câncer de pulmão^(7)^, assim como influenciam na distribuição do agravo no espaço^(2)^.

Tendo em vista o registro de câncer de forma capilarizada no território, o Ministério da Saúde do Brasil vem aprimorando as ações de prevenção, rastreamento, diagnóstico e tratamento sustentadas na Política Nacional para Prevenção e Controle do Câncer (PNPCC) e executadas na Rede de Atenção à Saúde (RAS) estruturada com sete componentes: Atenção Básica; Atenção Domiciliar; Atenção Especializada; Sistemas de Apoio; Regulação; Sistemas Logísticos; e Governança^(8)^, sendo que, na Atenção Básica, ocorre o rastreamento de casos dada a inserção das equipes de saúde nos territórios.

Portanto, para melhor compreender a relação do câncer com a dinâmica do espaço geográfico, identificaram-se as técnicas de análise espacial. Em Portugal, por exemplo, a regressão geograficamente ponderada (GWR) foi utilizada para investigar a relação entre o risco relativo de mortalidade por câncer de pulmão e a poluição do ar^(9)^, mostrando-se uma técnica robusta.

Não obstante, evidências de uma revisão de literatura que analisou a incidência de câncer de pulmão utilizando métodos de análise espacial referiram a não utilização de GWR para analisar a influência de indicadores socioeconômicos e ambientais sobre os casos novos do agravo, limitando os resultados obtidos. É válido mencionar que a utilização de modelos GWR permite a visualização do problema de forma mais detalhada, de acordo com cada região estudada, uma vez que aponta dependência espacial, quando for o caso^(10)^.

Diante dessa lacuna, este estudo tem como objetivo analisar a incidência de câncer de pulmão associada a indicadores sociais, econômicos e ambientais. A questão central consiste em compreender como esses determinantes influenciam a distribuição geográfica do agravo, partindo-se da hipótese de que há dependência espacial significativa entre os indicadores e a incidência do câncer de pulmão, o que pode revelar padrões territoriais relevantes para o planejamento em saúde.

## MÉTODO

### Desenho do Estudo

Trata-se de um estudo ecológico, com delineamento de grupos múltiplos.

### Contexto

O estado do Pará está situado na região Norte do Brasil, com uma população de 8.120.131 habitantes e área territorial de 1.245.870,704 km^2^. No ranking nacional, ocupa a 24ª posição no Índice de Desenvolvimento Humano (IDH), com valor de 0,646, e a 20ª posição no Índice de Vulnerabilidade Social (IVS), com valor de 0,299^(11)^. O estado é composto por 144 municípios, mas conta com apenas quatro estabelecimentos de saúde habilitados para oferecer assistência especializada e integral ao paciente com câncer, incluindo diagnóstico e tratamento^(12)^.

### Participantes

Foram incluídos no estudo 700 casos novos de câncer de pulmão, notificados entre 1º de janeiro de 2017 e 31 de dezembro de 2021. Todos os casos são procedentes de algum dos municípios do estado do Pará e foram obtidos por meio do sistema IntegradorRHC-INCA, que reúne dados hospitalares de registros de câncer.

### Variáveis

As variáveis analisadas foram agrupadas em categorias socioeconômicas, ambientais e de estrutura de saúde. Entre as variáveis socioeconômicas, destacam-se o Índice de Desenvolvimento Humano Municipal (IDHM), Produto Interno Bruto (PIB), IVS, Índice de Gini, renda per capita, taxa de analfabetismo, cobertura de atenção básica e proporção de pobres, extremamente pobres e vulneráveis à pobreza. As variáveis ambientais incluíram o Índice de Qualidade do Ar (IQA), porcentagem de cobertura vegetal natural, focos de calor e incremento de desmatamento. Também foram consideradas variáveis relacionadas ao mercado de trabalho, como a porcentagem de ocupados nos setores agropecuário, extrativo mineral, indústria de transformação, serviços industriais de utilidade pública, construção, comércio e serviços.

### Fonte de Dados/Mensuração

Os dados populacionais e cartográficos foram obtidos do Instituto Brasileiro de Geografia e Estatística (IBGE), incluindo a base dos 144 municípios do Estado. As taxas médias anuais de incidência de câncer de pulmão foram calculadas por município e ajustadas por faixa etária, utilizando o método direto^(13)^ com base no censo demográfico de 2022. O software Microsoft® Excel® para Microsoft 365 MSO foi utilizado para os cálculos. As variáveis socioeconômicas foram extraídas do Atlas do Desenvolvimento Humano no Brasil (AtlasBR) e da plataforma e-Gestor AB do Ministério da Saúde. As variáveis ambientais foram obtidas das plataformas TerraBrasilis, Programa Queimadas (INPE) e do aplicativo AccuWeather. A densidade de estabelecimentos de saúde habilitados para assistência oncológica foi calculada com base em dados do Instituto Nacional de Câncer (INCA).

### Viés

Por se tratar de um estudo ecológico baseado em dados secundários agregados, há limitações quanto à precisão dos registros individuais. A possibilidade de subnotificação, inconsistência nos dados ou ausência de informações específicas pode introduzir viés na análise. Além disso, a utilização de dados agregados por município impede inferências diretas sobre indivíduos.

### Tamanho do Estudo

O estudo contemplou 700 casos novos de câncer de pulmão registrados no estado do Pará ao longo de cinco anos.

### Variáveis Quantitativas

As taxas de incidência foram expressas por município e ajustadas por faixa etária. Os indicadores socioeconômicos e ambientais foram considerados em suas formas numéricas.

### Métodos Estatísticos

Foi realizada inicialmente a análise de colinearidade entre a variável dependente, taxa média anual de incidência de câncer de pulmão ajustada por idade, e as variáveis independentes por meio da correlação de Pearson, utilizando o software Minitab 22.1. Em seguida, procedeu-se à avaliação da multicolinearidade entre as variáveis independentes por meio do Fator de Inflação da Variância (VIF), também no Minitab 22.1. Valores de VIF superiores a 10 foram considerados indicativos de multicolinearidade significativa, sendo tais variáveis excluídas do modelo subsequente.

Em seguida, aplicou-se a técnica “stepwise” no software Geoda 1.14.0 para seleção das variáveis mais relevantes, resultando em um modelo de regressão linear de mínimos quadrados ordinários (OLS) com o menor Critério de Informação de Akaike (AIC). O modelo foi avaliado quanto ao valor do intercepto, aos coeficientes β locais, ao p-valor, ao coeficiente de determinação (R^2^) e ao coeficiente de determinação ajustado (R^2^ ajustado). As variáveis selecionadas foram então utilizadas na construção do modelo GWR, e os resultados foram representados por meio de mapas temáticos elaborados no software ArcGIS® 10.6.

### Aspectos Éticos

Por se tratar de um estudo com dados secundários de domínio público, não foi necessária apreciação por Comitê de Ética em Pesquisa.

## RESULTADOS

O estudo analisou 700 casos novos de câncer de pulmão. Na Tabela 1, apresenta-se a análise estatística dos indicadores socioeconômicos e ambientais a partir da correlação de Pearson, a qual variou de –0,211 a 0,265.

**Tabela 1:** Correlação entre indicadores socioeconômicos e ambientais e a incidência de câncer de pulmão ajustada por idade – Belém, PA, Brasil (2024).

Variável	Correlação de Pearson	p-valor
IDHM	0,264	0,001
Renda per capita	0,265	0,001
Cobertura de atenção básica	0,239	0,004
Porcentagem de cobertura vegetal natural	-0,211	0,011
Porcentagem dos ocupados no setor agropecuário	-0,171	0,041
Porcentagem dos ocupados nos setores de serviços industriais de utilidade pública	0,192	0,021
Porcentagem dos ocupados no setor de construção	0,160	0,056
Porcentagem dos ocupados no setor comércio	0,188	0,024
Porcentagem dos ocupados no setor de serviços	0,199	0,017
Proporção de pobres	-0,197	0,018
Proporção de extremamente pobres	-0,191	0,022
Proporção de vulneráveis à pobreza	-0,174	0,037
Porcentagem dos ocupados na indústria de transformação	0,057	0,497
Porcentagem dos ocupados no setor extrativo mineral	-0,022	0,790
Taxa de analfabetismo	-0,149	0,074
Focos de calor	0,078	0,353
Índice de Gini	0,018	0,828
Incremento do desmatamento	-0,047	0,576
IQA	-0,040	0,631
IVS	0,014	0,872
PIB	0,088	0,294
Densidade de estabelecimentos habilitados	0,144	0,085

Em relação à variável dependente, taxa média anual de incidência de câncer de pulmão ajustada, as variáveis IDHM, renda per capita, cobertura de atenção básica, porcentagem de cobertura vegetal natural, porcentagem dos ocupados no setor agropecuário, porcentagem dos ocupados nos setores de serviços industriais de utilidade pública, porcentagem dos ocupados no setor de construção, porcentagem dos ocupados no setor comércio, porcentagem dos ocupados no setor de serviços, proporção de pobres e proporção de vulneráveis à pobreza apresentaram significância estatística. A análise de multicolinearidade por meio do VIF indicou que as variáveis renda per capita e cobertura de atenção básica apresentaram valores inferiores ao ponto de corte de 10, não evidenciando multicolinearidade significativa entre os preditores e permitindo a inclusão destas no modelo seguinte.

O modelo OLS apresentou R2 ajustado que explicou apenas 9,93% da variabilidade das taxas médias anuais, de incidência de câncer de pulmão ajustadas por idade, no período. Como variáveis preditoras, permaneceram renda per capita e cobertura de atenção básica (Tabela 2).

**Tabela 2:** Modelo OLS para a incidência de câncer de pulmão ajustada por idade – Belém, PA, Brasil (2024).

Variáveis	Estimativa	Erro padrão	p-Valor
Constante	–0,0495123	0,0746433	0,50821
Renda per capita	0,000476318	0,00016111	0,00365
Cobertura de atenção básica	0,00208483	0,000806072	0,01071

R^2^ = 0,111; R^2^ ajustado = 0,099.

p-Valor do modelo < 0,001.

Observa-se que a estimativa do coeficiente para a renda per capita e para a cobertura de atenção básica é positiva e estatisticamente significativa, sugerindo que um aumento nessas duas variáveis está associado à elevação nas taxas de incidência de câncer de pulmão.

Após a definição do modelo OLS, recorreu-se à GWR, utilizando uma banda fixa para melhor ajuste ao modelo, confirmado pelo menor AIC (banda fixa = –7,684; banda adaptativa = –7,385). Com este modelo, foram obtidos um R^2^ = 0,136 e um R^2^ ajustado = 0,107, tendo o R^2^ local uma variação de 0,09 a 0,13 (Figura 1).

**Figura 1:** Distribuição espacial do R^2^ local da regressão geograficamente ponderada para a incidência de câncer de pulmão ajustada por idade – Belém, PA, Brasil (2024).

A Figura 2 apresenta a distribuição espacial dos coeficientes β locais das variáveis independentes que compuseram o modelo. Os coeficientes β locais para a renda per capita indicam variações consideráveis em diferentes regiões, com áreas no Sudoeste e Sudeste Paraense apresentando coeficientes positivos mais elevados, sugerindo uma associação mais forte entre a renda per capita e a taxa de incidência nessas localidades.

**Figura 2:** Distribuição espacial dos coeficientes β locais das variáveis independentes na regressão geograficamente ponderada para a incidência de câncer de pulmão ajustada por idade – Belém, PA, Brasil (2024).

Por outro lado, os coeficientes β locais da variável “cobertura de atenção básica” demonstraram que em mesorregiões, como Baixo Amazonas, Marajó e Metropolitana de Belém, a relação é mais pronunciada, refletindo maior influência na incidência do câncer de pulmão em razão da oportunidade de maior acesso ao diagnóstico.

## DISCUSSÃO

O presente estudo evidenciou que a incidência de câncer de pulmão é autocorrelacionada no estado do Pará, sendo a renda per capita uma das preditoras para a ocorrência do evento.

Resultado semelhante foi observado em outro estudo brasileiro, que analisou o comportamento do câncer de pulmão em centros urbanos, identificando associação positiva com a renda per capita tanto para o diagnóstico em estágio avançado quanto para a mortalidade por câncer de pulmão, sendo esta relação evidenciada em territórios com os maiores índices de desenvolvimento, com população mais envelhecida e melhor oferta de serviços de saúde^(14)^.

Isto ocorre porque a renda per capita tem forte influência sobre o estilo de vida relativo à promoção da saúde evidenciada nas práticas de atividades físicas, autogestão, nutrição e responsabilidade pela saúde^(15)^, repercutindo em maior expectativa de vida. Outrossim, com maior renda, há mais investimentos em educação em saúde, possível redução dos riscos do tabagismo e reconhecimento da importância de realização de exames regulares, contribuindo para a detecção precoce do câncer de pulmão^(16)^.

É válido mencionar ainda que em territórios com maior renda per capita, há melhor acesso a serviços de saúde, incluindo diagnóstico precoce e tratamento avançado^(17)^. Destaca-se aqui a Atenção Básica, ou Atenção Primária à Saúde (APS), cujas variações positivas em sua cobertura, neste estudo, também foram identificadas como preditoras para o aumento da incidência de câncer de pulmão no estado do Pará.

Dado o desenvolvimento socioeconômico, emerge a necessidade de melhor coordenação dos cuidados à saúde. Nesse contexto, a atenção básica, como o primeiro nível de atenção, atua como coordenadora e ordenadora da RAS, fornecendo cuidados preventivos e intervenções para gerenciar a carga de DCNT, entre elas o câncer de pulmão. Como a porta de entrada preferencial à RAS, a APS tem um papel crítico no diagnóstico precoce e no encaminhamento para os outros níveis de atenção^(18)^.

Nesse sentido, nos Estados Unidos, a U.S. Preventive Services Task Force (USPSTF) recomenda que pacientes entre 50 e 80 anos, com histórico de tabagismo de 20 anos, que fumam atualmente ou pararam de fumar nos últimos 15 anos, façam anualmente o exame de tomografia computadorizada de baixa dose (LDCT) para câncer de pulmão^(19)^. Contudo, cerca de 50% dos elegíveis para LDCT, de acordo com as recomendações do USPSTF, não têm seguro ou são segurados pelo Medicaid, que, em muitos Estados, não cobre o exame^(20,21)^.

Ademais, ainda no país supracitado, existem outros obstáculos para o diagnóstico de câncer de pulmão^(19)^. Residentes de áreas rurais, embora mais afetados pelo tabagismo e pela incidência do agravo em relação às áreas urbanas, tendem a ter cobertura de seguro inadequada e enfrentam barreiras geográficas de acesso^(22,23)^. Dessa forma, são mais propensos a ter acesso limitado a profissionais de atenção primária, que recomendariam a LDCT e encaminhariam a cuidados especializados, quando necessário^(24)^.

Nesse contexto dos EUA, fora identificado que a baixa cobertura do seguro de saúde refletiu no rastreamento do câncer de pulmão, assim como no diagnóstico de asma e outras doenças respiratórias. A ampliação do rastreio recomendado foi significativa entre os homens de alto risco, mas não entre as mulheres com o mesmo risco, sendo lacunas na conscientização dos pacientes sobre a opção de serem examinados, conhecimento dos benefícios do exame, estigma relacionado ao tabagismo e falta de acesso ao atendimento dos fatores contribuintes para a baixa adesão feminina^(25)^.

No Pará, ainda que existam desafios significativos para o alcance da cobertura ideal, entre os anos de 2010 e 2021 houve um crescimento progressivo na cobertura da APS por meio da Estratégia Saúde da Família (ESF)^(26)^. Este fato pode justificar os achados deste estudo, uma vez que, com uma cobertura de atenção básica mais ampla, há mais oportunidades para a detecção precoce de doenças e acesso a exames diagnósticos para o câncer de pulmão.

Considerando a modalidade deste estudo reconhece-se a falácia ecológica como possível limitação da pesquisa, não podendo os resultados serem considerados em nível individual. Além do mais, há utilização de dados secundários, o que pode induzir a presença de viés de informação pela qualidade do preenchimento dos dados. Em adição, a complexidade do modelo GWR pode implicar em dificuldades na sua aplicação e interpretação.

Todavia, a aplicação da regressão espacial geograficamente ponderada permitiu uma visão mais detalhada e localizada das relações entre as variáveis, visto que ao contrário dos modelos de regressão tradicionais, considera a variação geográfica local, uma vez que um padrão aplicado a uma área não se aplica necessariamente às demais^(27)^. Para a enfermagem, isso representa uma oportunidade de atuação mais estratégica, com foco em territórios prioritários e populações vulneráveis, promovendo equidade no acesso e na qualidade do cuidado.

Por fim, os achados reforçam a importância da articulação entre vigilância epidemiológica, gestão territorial e práticas clínicas. A enfermagem, como força de trabalho majoritária no Sistema Único de Saúde, está posicionada para liderar iniciativas de prevenção, educação e cuidado integral, contribuindo para a redução da morbimortalidade por câncer de pulmão e para o fortalecimento da rede de atenção à saúde.

## CONCLUSÃO

Os resultados deste estudo evidenciam que, no estado do Pará, em especial na região metropolitana de Belém, a incidência de câncer de pulmão reflete diretamente o desenvolvimento socioeconômico e a estruturação da rede de assistência em saúde. A renda per capita mais elevada e a maior cobertura da APS associaram-se ao aumento da detecção da doença, indicando que o diagnóstico precoce e o acesso a especialistas são viabilizados em territórios com melhores condições socioeconômicas e maior organização da rede de atenção.

Dessa forma, torna-se essencial direcionar intervenções e recursos estratégicos para ampliar a equidade, fortalecendo a APS como porta de entrada para prevenção, rastreamento e encaminhamento oportuno. Ao expandir a cobertura e qualificar os serviços, será possível reduzir desigualdades regionais e promover maior impacto na morbimortalidade por câncer de pulmão.

Revela-se uma base sólida para futuras pesquisas aprofundarem as repercussões da APS na prevenção e controle do câncer de pulmão, em especial estudos que demonstrem o impacto econômico da doença e maneiras eficazes de utilização dos recursos.

## Data Availability

Todo o conjunto de dados que dá suporte aos resultados deste estudo foi publicado no próprio artigo.

# Translation: El cáncer de pulmón en el contexto socioeconómico y ambiental de la Amazonía de Pará

**DOI:** 10.1590/1980-220X-REEUSP-2026-0036es

**Authors:** Bruna Rafaela Leite Dias, Laura Maria Vidal Nogueira, Ivaneide Leal Ataíde Rodrigues, Eliã Pinheiro Botelho, Bruna Puty, Maridalva Ramos Leite, Gracileide Maia Corrêa, Altem Nascimento Pontes

**Affiliations:** 1 Universidade do Estado do Pará, Centro de Ciências Biológicas e da Saúde, Programa de Pós-Graduação em Enfermagem, Belém, PA, Brasil.; 2 Universidade Federal do Pará, Instituto de Ciências da Saúde, Programa de Pós-Graduação em Enfermagem, Belém, PA, Brasil.; 3 Faculdade Integrada da Amazônia, Belém, PA, Brasil.; 4 Universidade do Estado do Pará, Centro de Ciências Biológicas e da Saúde, Departamento de Enfermagem Comunitária, Belém, PA, Brasil.; 5 Universidade do Estado do Pará, Centro de Ciências Naturais e Tecnologia, Programa de Pós-Graduação em Ciências Ambientais, Belém, PA, Brasil.

**Keywords:** Enfermedades no Transmisibles, Neoplasias Pulmonares, Análisis Espacial

## Abstract

**Objetivo:**

Analizar la incidencia del cáncer de pulmón asociada a indicadores sociales, económicos y ambientales.

**Método:**

Se trata de un estudio ecológico con 700 nuevos casos de cáncer de pulmón, extraídos de la base de datos IntegradorRHC-INCA. Se calcularon las tasas de incidencia anuales promedio, se ajustaron mediante el método directo y se sometieron a un análisis de regresión ponderada geográficamente. Las variables independientes fueron indicadores socioeconómicos y ambientales, incluyendo el ingreso per cápita y la cobertura de atención primaria de salud.

**Resultados:**

Se observó una correlación positiva entre la incidencia y el ingreso per cápita, así como con la cobertura de atención primaria de salud.

**Conclusión:**

Los resultados ponen de manifiesto la necesidad de intervenciones específicas y la asignación estratégica de recursos en áreas prioritarias, así como la ampliación y el fortalecimiento de la Atención Primaria de Salud para la detección precoz, la prevención y la orientación sobre los riesgos de desarrollar cáncer de pulmón.

## INTRODUCCIÓN

El cáncer es un importante problema de salud pública, que presenta una fuerte relación con factores socioeconómicos, sobre todo en el siglo XXI, siendo responsable de aproximadamente una de cada seis muertes prematuras (16,8 %) y una de cada cuatro muertes (22,8 %) por enfermedades crónicas no transmisibles (ECNT) en todo el mundo^(1)^. Con casi 2,5 millones de casos nuevos y más de 1,8 millones de muertes en todo el mundo, el cáncer de pulmón fue la principal causa de morbilidad y mortalidad por cáncer en 2022, ocupando el primer lugar entre los hombres y el segundo entre las mujeres^(2)^.

En este contexto, en Brasil, el cáncer de pulmón fue el más mortal en 2020, y se estima que será uno de los más frecuentes en el período de 2023 a 2025, al igual que los cánceres de mama y de próstata^(3)^. Además, según las previsiones de las tasas de incidencia global estandarizadas por edad, se cree que estas seguirán aumentando drásticamente entre las mujeres en la mayoría de los países hasta 2035, incluido Brasil^(4)^.

Desde esta perspectiva, el número previsto de nuevos casos de cáncer de tráquea, bronquios y pulmón en Brasil, para cada año del trienio de 2023 a 2025, es de 32 560 casos, lo que corresponde a un riesgo estimado de 15,06 casos por cada 100 000 habitantes en el año 2023.

En el mismo período, el estado de Pará, territorio de la Amazonía Legal, presentó una tasa de incidencia de 7,25 casos por cada 100 000 habitantes^(5)^.

Destacan los determinantes sociales de la salud, considerados factores que influyen directamente en los niveles de salud de la población, incluyendo vivienda, saneamiento básico, medio ambiente, trabajo e ingresos, entre otros^(6)^. Estos determinantes, a su vez, están asociados al riesgo de enfermedad y a los resultados del tratamiento del cáncer de pulmón^(7)^, así como influyen en la distribución de la enfermedad en el espacio^(2)^.

Teniendo en cuenta el registro ampliamente distribuido del cáncer en el territorio, el Ministerio de Salud de Brasil ha venido mejorando las acciones de prevención, detección, diagnóstico y tratamiento sustentadas en la Política Nacional para la Prevención y el Control del Cáncer (PNPCC) y ejecutadas en la Red de Atención a la Salud (RAS), estructurada en siete componentes: Atención Primaria; Atención Domiciliaria; Atención Especializada; Sistemas de Apoyo; Regulación; Sistemas Logísticos; y Gobernanza^(8)^, y es en la Atención Básica donde se lleva a cabo el rastreo de casos, dada la presencia de los equipos de salud en los territorios.

Por lo tanto, para comprender mejor la relación del cáncer con la dinámica del espacio geográfico, se identificaron las técnicas de análisis espacial. En Portugal, por ejemplo, se utilizó la regresión geográficamente ponderada (GWR) para investigar la relación entre el riesgo relativo de mortalidad por cáncer de pulmón y la contaminación del aire^(9)^, demostrándose que es una técnica robusta.

No obstante, las evidencias de una revisión bibliográfica que analizó la incidencia del cáncer de pulmón utilizando métodos de análisis espacial señalaron la no utilización de la GWR para analizar la influencia de indicadores socioeconómicos y ambientales sobre los nuevos casos de la enfermedad, lo que limitó los resultados obtenidos. Cabe mencionar que el uso de modelos GWR permite visualizar el problema de manera más detallada, de acuerdo con cada región estudiada, ya que señala la dependencia espacial, cuando sea el caso^(10)^.

Ante esta laguna de conocimiento, este estudio tiene como objetivo analizar la incidencia del cáncer de pulmón asociada a indicadores sociales, económicos y ambientales. La cuestión central consiste en comprender cómo estos determinantes influyen en la distribución geográfica de la enfermedad, partiendo de la hipótesis de que existe una dependencia espacial significativa entre los indicadores y la incidencia del cáncer de pulmón, lo que puede revelar patrones territoriales relevantes para la planificación en salud.

## MÉTODO

### Diseño del Estudio

Se trata de un estudio ecológico, con un diseño de grupos múltiples.

### Contexto

El estado de Pará está situado en la región Norte de Brasil, con una población de 8 120 131 habitantes y una superficie territorial de 1 245 870,704 km^2^. En el ranking nacional, ocupa el puesto 24 en el Índice de Desarrollo Humano (IDH), con un valor de 0,646, y el puesto 20 en el Índice de Vulnerabilidad Social (IVS), con un valor de 0,299^(11)^.

El estado está compuesto por 144 municipios, pero cuenta con solo cuatro centros de salud habilitados para ofrecer atención especializada e integral al paciente con cáncer, incluyendo diagnóstico y tratamiento^(12)^.

### Participantes

Se incluyeron en el estudio 700 casos nuevos de cáncer de pulmón, notificados entre el 1 de enero de 2017 y el 31 de diciembre de 2021. Todos los casos proceden de alguno de los municipios del estado de Pará y se obtuvieron a través del sistema IntegradorRHC-INCA, que recopila datos hospitalarios de registros de cáncer.

### Variables

Las variables analizadas se agruparon en categorías socioeconómicas, ambientales y de estructura de salud. Entre las variables socioeconómicas, destacan el Índice de Desarrollo Humano Municipal (IDHM), el Producto Interno Bruto (PIB), el IVS, el Índice de Gini, el ingreso per cápita, la tasa de analfabetismo, la cobertura de atención primaria y la proporción de personas pobres, extremadamente pobres y vulnerables a la pobreza. Las variables ambientales incluyeron el Índice de Calidad del Aire (IQA), el porcentaje de cobertura vegetal natural, los focos de calor y el incremento de la deforestación. También se consideraron variables relacionadas con el mercado laboral, como el porcentaje de personas ocupadas en los sectores agropecuario, extractivo mineral, industria de transformación, servicios industriales de utilidad pública, construcción, comercio y servicios.

### Fuente de Datos/Medición

Los datos poblacionales y cartográficos se obtuvieron del Instituto Brasileño de Geografía y Estadística (IBGE), incluyendo la base de los 144 municipios del estado. Las tasas medias anuales de incidencia de cáncer de pulmón se calcularon por municipio y se ajustaron por grupo de edad, utilizando el método directo^(13)^ basado en el censo demográfico de 2022. Se utilizó el software Microsoft® Excel® para Microsoft 365 MSO para los cálculos. Las variables socioeconómicas se extrajeron del Atlas del Desarrollo Humano en Brasil (AtlasBR) y de la plataforma e-Gestor AB del Ministerio de Salud. Las variables ambientales se obtuvieron de las plataformas TerraBrasilis, Programa Queimadas (INPE) y de la aplicación AccuWeather. La densidad de centros de salud habilitados para la atención oncológica se calculó a partir de datos del Instituto Nacional del Cáncer (INCA).

### Sesgo

Al tratarse de un estudio ecológico basado en datos secundarios agregados, existen limitaciones en cuanto a la precisión de los registros individuales. La posibilidad de subnotificación, inconsistencia en los datos o ausencia de información específica puede introducir sesgos en el análisis. Además, el uso de datos agregados por municipio impide realizar inferencias directas sobre individuos.

### Tamaño del Estudio

El estudio incluyó 700 casos nuevos de cáncer de pulmón registrados en el estado de Pará a lo largo de cinco años.

### Variables Cuantitativas

Las tasas de incidencia se expresaron por municipio y se ajustaron por grupo de edad. Los indicadores socioeconómicos y ambientales se consideraron en sus formas numéricas.

### Métodos Estadísticos

Inicialmente se realizó el análisis de colinealidad entre la variable dependiente, la tasa media anual de incidencia de cáncer de pulmón ajustada por edad, y las variables independientes mediante la correlación de Pearson, utilizando el software Minitab 22.1. A continuación, se procedió a la evaluación de la multicolinealidad entre las variables independientes mediante el Factor de Inflamación de la Varianza (VIF), también en Minitab 22.1. Los valores de VIF superiores a 10 se consideraron indicativos de multicolinealidad significativa, por lo que dichas variables se excluyeron del modelo posterior.

A continuación, se aplicó la técnica «stepwise» en el software Geoda 1.14.0 para la selección de las variables más relevantes, lo que dio como resultado un modelo de regresión lineal de mínimos cuadrados ordinarios (OLS) con el menor Criterio de Información de Akaike (AIC). El modelo se evaluó en cuanto al valor de la intersección, los coeficientes β locales, el valor p, el coeficiente de determinación (R^2^) y el coeficiente de determinación ajustado (R^2^ ajustado). Las variables seleccionadas se utilizaron entonces en la construcción del modelo GWR, y los resultados se representaron mediante mapas temáticos elaborados en el software ArcGIS® 10.6.

### Aspectos Éticos

Al tratarse de un estudio con datos secundarios de dominio público, no fue necesaria la evaluación por parte de un Comité de Ética en Investigación.

## RESULTADOS

El estudio analizó 700 casos nuevos de cáncer de pulmón. En la Tabla 1 se presenta el análisis estadístico de los indicadores socioeconómicos y ambientales a partir de la correlación de Pearson, la cual varió de –0,211 a 0,265.

**Tabla 1:** Correlación entre indicadores socioeconómicos y ambientales y la incidencia de cáncer de pulmón ajustada por edad – Belém, PA, Brasil (2024).

Variable	Correlación de Pearson	Valor p
IDHM	0,264	0,001
Renta per cápita	0,265	0,001
Cobertura de atención primaria	0,239	0,004
Porcentaje de cobertura vegetal natural	-0,211	0,011
Porcentaje de personas empleadas en el sector agrícola	-0,171	0,041
Porcentaje de personas ocupadas en los sectores de servicios industriales de utilidad pública	0,192	0,021
Porcentaje de personas ocupadas en el sector de la construcción	0,160	0,056
Porcentaje de personas ocupadas en el sector del comercio	0,188	0,024
Porcentaje de personas ocupadas en el sector de los servicios	0,199	0,017
Proporción de personas en situación de pobreza	-0,197	0,018
Proporción de personas en situación de pobreza extrema	-0,191	0,022
Proporción de personas vulnerables a la pobreza	-0,174	0,037
Porcentaje de ocupados en la industria manufacturera	0,057	0,497
Porcentaje de personas ocupadas en el sector extractivo mineral	-0,022	0,790
Tasa de analfabetismo	-0,149	0,074
Puntos calientes	0,078	0,353
Índice de Gini	0,018	0,828
Aumento de la deforestación	-0,047	0,576
IQA	-0,040	0,631
IVS	0,014	0,872
PIB	0,088	0,294
Densidad de establecimientos autorizados	0,144	0,085

En relación con la variable dependiente, la tasa media anual ajustada de incidencia de cáncer de pulmón, las variables IDHM, la renta per cápita, la cobertura de atención primaria, el porcentaje de cobertura vegetal natural, el porcentaje de personas ocupadas en el sector agropecuario, el porcentaje de personas ocupadas en los sectores de servicios industriales de utilidad pública, el porcentaje de personas ocupadas en el sector de la construcción, el porcentaje de personas ocupadas en el sector comercial, el porcentaje de personas ocupadas en el sector de servicios, la proporción de personas pobres y la proporción de personas vulnerables a la pobreza presentaron significación estadística. El análisis de multicolinealidad mediante el VIF indicó que las variables renta per cápita y cobertura de atención primaria presentaron valores inferiores al umbral de 10, lo que no evidenció una multicolinealidad significativa entre los predictores y permitió su inclusión en el siguiente modelo.

El modelo OLS presentó un R^2^ ajustado que explicaba solo el 9,93 % de la variabilidad de las tasas medias anuales de incidencia de cáncer de pulmón ajustadas por edad en el periodo. Como variables predictivas se mantuvieron la renta per cápita y la cobertura de atención primaria (Tabla 2).

**Tabla 2:** Modelo OLS para la incidencia de cáncer de pulmón ajustada por edad – Belém, PA, Brasil (2024).

Variables	Estimación	Error estándar	Valor p
Constante	–0,0495123	0,0746433	0,50821
Renta per cápita	0,000476318	0,00016111	0,00365
Cobertura de atención primaria	0,00208483	0,000806072	0,01071

R^2^ = 0,111; R^2^ ajustado = 0,099.

Valor p del modelo < 0,001.

Se observa que la estimación del coeficiente para la renta per cápita y para la cobertura de atención primaria es positiva y estadísticamente significativa, lo que sugiere que un aumento en estas dos variables se asocia con un incremento en las tasas de incidencia del cáncer de pulmón.

Tras definir el modelo OLS, se recurrió al GWR, utilizando un intervalo fijo para un mejor ajuste al modelo, lo cual se confirmó por el menor AIC (intervalo fijo = –7,684; intervalo adaptativo = –7,385). Con este modelo, se obtuvieron un R^2^ = 0,136 y un R^2^ ajustado = 0,107, con un R^2^ local que varió de 0,09 a 0,13 (Figura 1).

**Figura 1:** Distribución espacial del R2 local de la regresión ponderada geográficamente para la incidencia de cáncer de pulmón ajustada poredad – Belém, PA, Brasil (2024).

La Figura 2 presenta la distribución espacial de los coeficientes β locales de las variables independientes que compusieron el modelo. Los coeficientes β locales para el ingreso per cápita indican variaciones considerables en diferentes regiones, con áreas en el suroeste y sureste de Pará que presentan coeficientes positivos más elevados, lo que sugiere una asociación más fuerte entre el ingreso per cápita y la tasa de incidencia en esas localidades.

**Figura 2:** Distribución espacial de los coeficientes β locales de las variables independientes en la regresión ponderada geográficamente para la incidencia de cáncer de pulmón ajustada por edad – Belém, PA, Brasil (2024).

Por otro lado, los coeficientes β locales de la variable «cobertura de atención primaria» demostraron que en mesorregiones, como el Bajo Amazonas, Marajó y la Metropolitana de Belém, la relación es más pronunciada, lo que refleja una mayor influencia en la incidencia del cáncer de pulmón debido a la oportunidad de un mayor acceso al diagnóstico.

## DISCUSIÓN

El presente estudio evidenció que la incidencia del cáncer de pulmón está autocorrelacionada en el estado de Pará, siendo el ingreso per cápita uno de los predictores de la ocurrencia del evento.

Se observó un resultado similar en otro estudio brasileño, que analizó el comportamiento del cáncer de pulmón en centros urbanos, identificando una asociación positiva con la renta per cápita tanto para el diagnóstico en estadio avanzado como para la mortalidad por cáncer de pulmón, relación que se evidenció en territorios con los mayores índices de desarrollo, con una población más envejecida y una mejor oferta de servicios de salud^(14)^.

Esto ocurre porque la renta per cápita tiene una fuerte influencia sobre el estilo de vida relacionado con la promoción de la salud, evidenciado en las prácticas de actividad física, autogestión, nutrición y responsabilidad por la salud^(15)^, lo que repercute en una mayor esperanza de vida. Además, con mayores ingresos, hay más inversiones en educación en salud, una posible reducción de los riesgos del tabaquismo y el reconocimiento de la importancia de realizarse exámenes regulares, lo que contribuye a la detección temprana del cáncer de pulmón^(16)^.

Cabe mencionar también que en los territorios con mayor ingreso per cápita, hay mejor acceso a los servicios de salud, incluyendo el diagnóstico temprano y el tratamiento avanzado^(17)^. Cabe destacar aquí la Atención Básica, o Atención Primaria de Salud (APS), cuyas variaciones positivas en su cobertura, en este estudio, también se identificaron como predictoras del aumento de la incidencia de cáncer de pulmón en el estado de Pará.

Dado el desarrollo socioeconómico, surge la necesidad de una mejor coordinación de la atención de salud. En este contexto, la atención básica, como primer nivel de atención, actúa como coordinadora y organizadora del RAS, proporcionando cuidados preventivos e intervenciones para gestionar la carga de las ENT, entre ellas el cáncer de pulmón. Como puerta de entrada preferencial al RAS, la APS tiene un papel crítico en el diagnóstico precoz y en la derivación a otros niveles de atención^(18)^.

En este sentido, en los Estados Unidos, el Grupo de Trabajo de Servicios Preventivos de los Estados Unidos (USPSTF) recomienda que los pacientes de entre 50 y 80 años, con un historial de tabaquismo de 20 años, que fuman actualmente o dejaron de fumar en los últimos 15 años, se sometan anualmente a una tomografía computarizada de baja dosis (LDCT) para detectar el cáncer de pulmón^(19)^. Sin embargo, alrededor del 50 % de las personas elegibles para la LDCT, según las recomendaciones de la USPSTF, no tienen seguro o están aseguradas por Medicaid, que, en muchos estados, no cubre el examen^(20,21)^.

Además, en el país mencionado anteriormente, existen otros obstáculos para el diagnóstico del cáncer de pulmón^(19)^. Los residentes de zonas rurales, aunque se ven más afectados por el tabaquismo y la incidencia de la enfermedad en comparación con las zonas urbanas, tienden a tener una cobertura de seguro inadecuada y enfrentan barreras geográficas de acceso^(22,23)^. De este modo, son más propensos a tener acceso limitado a profesionales de atención primaria, quienes recomendarían la TAC de baja dosis y derivarían a cuidados especializados, cuando sea necesario^(24)^.

En este contexto de los EE. UU., se identificó que la baja cobertura del seguro médico se reflejó en el cribado del cáncer de pulmón, así como en el diagnóstico de asma y otras enfermedades respiratorias. La ampliación del cribado recomendado fue significativa entre los hombres de alto riesgo, pero no entre las mujeres con el mismo riesgo, siendo las lagunas en la concienciación de las pacientes sobre la opción de someterse a la prueba, el conocimiento de los beneficios de la misma, el estigma relacionado con el tabaquismo y la falta de acceso a la atención médica los factores que contribuyeron a la baja participación femenina^(25)^.

En Pará, aunque existen desafíos significativos para alcanzar la cobertura ideal, entre los años 2010 y 2021 hubo un crecimiento progresivo en la cobertura de la APS a través de la Estrategia de Salud Familiar (ESF)^(26)^. Este hecho puede justificar los hallazgos de este estudio, ya que, con una cobertura de atención primaria más amplia, hay más oportunidades para la detección temprana de enfermedades y el acceso a exámenes diagnósticos para el cáncer de pulmón.

Teniendo en cuenta la modalidad de este estudio, se reconoce la falacia ecológica como una posible limitación de la investigación, por lo que los resultados no pueden considerarse a nivel individual. Además, se utilizan datos secundarios, lo que puede dar lugar a un sesgo de información debido a la calidad del llenado de los datos. Por otra parte, la complejidad del modelo GWR puede implicar dificultades en su aplicación e interpretación.

No obstante, la aplicación de la regresión espacial ponderada geográficamente permitió una visión más detallada y localizada de las relaciones entre las variables, ya que, a diferencia de los modelos de regresión tradicionales, considera la variación geográfica local, dado que un patrón aplicado a un área no se aplica necesariamente a las demás^(27)^. Para la enfermería, esto representa una oportunidad de actuación más estratégica, centrada en territorios prioritarios y poblaciones vulnerables, promoviendo la equidad en el acceso y la calidad de la atención.

Por último, los hallazgos refuerzan la importancia de la articulación entre la vigilancia epidemiológica, la gestión territorial y las prácticas clínicas. La enfermería, como fuerza laboral mayoritaria en el Sistema Único de Salud, está posicionada para liderar iniciativas de prevención, educación y atención integral, contribuyendo a la reducción de la morbilidad y mortalidad por cáncer de pulmón y al fortalecimiento de la red de atención de salud.

## CONCLUSIÓN

Los resultados de este estudio evidencian que, en el estado de Pará, especialmente en la región metropolitana de Belém, la incidencia del cáncer de pulmón refleja directamente el desarrollo socioeconómico y la estructuración de la red de asistencia en salud. La renta per cápita más elevada y la mayor cobertura de la APS se asociaron con un aumento en la detección de la enfermedad, lo que indica que el diagnóstico precoz y el acceso a especialistas son viables en territorios con mejores condiciones socioeconómicas y una mayor organización de la red de atención.

De este modo, resulta esencial orientar las intervenciones y los recursos estratégicos para ampliar la equidad, fortaleciendo la APS como puerta de entrada para la prevención, el cribado y la derivación oportuna. Al ampliar la cobertura y mejorar la calidad de los servicios, será posible reducir las desigualdades regionales y promover un mayor impacto en la morbilidad y mortalidad por cáncer de pulmón.

Se revela una base sólida para que futuras investigaciones profundicen en las repercusiones de la APS en la prevención y el control del cáncer de pulmón, en especial estudios que demuestren el impacto económico de la enfermedad y las formas eficaces de utilizar los recursos.

## Data Availability

Todo el conjunto de datos que respalda los resultados de este estudio se publicó en el propio artículo.
